# Beyond DSM Categories: Criteria for Biologically Valid Disease Axes in Psychiatry

**DOI:** 10.3390/jcm15124830

**Published:** 2026-06-22

**Authors:** Lukasz Szarpak, Bernard Rybczynski, Michal Pruc, Bartosz W. Maj, Maciej Maslyk, Iwona Niewiadomska, Wieslaw J. Cubala

**Affiliations:** 1 Institute of Medical Science, The John Paul II Catholic University of Lublin, 20-708 Lublin, Poland; michal.pruc@kul.pl; 2 6th Department of Psychiatry, Mazovian Specialist Health Centre in Pruszkow, 05-802 Pruszkow, Poland; bernard.rybczynski@mscz.pl; 3 Department of Psychiatry, Health Center of Tomaszow Mazowiecki, 97-200 Tomaszow Mazowiecki, Poland; 4 Institute of Molecular Biology, The John Paul II Catholic University of Lublin, 20-708 Lublin, Poland; 5 Institute of Psychology, The John Paul II Catholic University of Lublin, 20-950 Lublin, Poland; iwona.niewiadomska@kul.pl; 6 Department of Psychiatry, Faculty of Medicine, Medical University of Gdansk, 80-211 Gdansk, Poland; cubala@gumed.edu.pl

**Keywords:** psychiatric nosology, biological validity, disease axes, dimensional psychopathology, transdiagnostic spectra, p factor, RDoC

## Abstract

Dimensional and transdiagnostic models have become central to contemporary efforts to move psychiatric nosology beyond DSM/ICD categories. This shift reflects persistent limitations of categorical syndromes as final biological targets, including within-diagnosis heterogeneity, cross-diagnostic comorbidity, developmental instability, and incomplete alignment with underlying mechanisms. This article examines a central unresolved problem in this transition: when, if ever, a descriptive or predictive psychiatric dimension can be interpreted as a candidate disease axis. We conducted a conceptual synthesis of major dimensional and transdiagnostic frameworks, including Research Domain Criteria (RDoC), Hierarchical Taxonomy of Psychopathology (HiTOP), the general psychopathology factor, cross-disorder genomic models, clinical staging approaches, and data-driven subtyping. The analysis separates three levels of inference that are often conflated in psychiatric research: descriptive structure, predictive utility, and disease-level biological validity. The synthesis identifies a recurrent inferential error in which reproducible factors, clusters, or classifiers are prematurely treated as evidence of disease architecture. Such constructs may describe real covariance patterns or improve prognostic prediction without establishing biological validity. We propose an eight-domain hierarchical framework for promotion to candidate disease-axis status, organized into four core gatekeepers—replication across cohorts, ascertainment, and methods, developmental coherence, incremental prognostic value beyond diagnosis and nonspecific severity, and discriminability from nonspecific severity—and four supporting/disciplining domains: cross-level convergence, mechanistic constraint, clinical leverage, and explicit falsifiability/boundary conditions. On this basis, middle-level transdiagnostic spectra and selected cross-disorder genomic liabilities appear more defensible as candidate disease axes than highly global or weakly specified constructs. Psychiatry was justified in turning toward dimensional models, but dimensionality alone does not confer biological validity. The key task is not to choose between categories and dimensions, but to define the evidential thresholds under which dimensional constructs warrant ontological promotion.

## 1. Introduction

Psychiatric diagnosis has always served two related but non-identical purposes: clinical coordination and scientific explanation. The syndromic tradition was not a conceptual mistake. It was a pragmatic response to a field that lacked pathognomonic lesions, laboratory assays, and etiologically settled disease entities [1,2]. Robins and Guze’s formulation remains instructive for exactly that reason: it treated diagnosis as a disciplined hypothesis about disease, not as disease itself [1,2].

That distinction became easier to forget as the *Diagnostic and Statistical Manual of Mental Disorders* (DSM) and international classification of diseases (ICD) matured. Their gains were substantial. They improved reliability, stabilized communication, enabled epidemiology and trials, and gave psychiatry a workable clinical language [3,4]. But, as Kendell and Jablensky emphasized, utility and validity are not the same thing [3]. A category may be clinically useful, prognostically informative, and indispensable for practice while still failing to correspond to a discrete biological kind [4].

DSM-III crystallized that bargain. Operational criteria improved reliability, but they also encouraged reification [5]. Hyman’s point remains central: once checklist-defined syndromes become the default units of inquiry, they begin to function as though their boundaries were natural [6]. Biology is then asked to validate constructs that biology did not create [4]. When findings are inconsistent, the failure is too easily attributed to immature methods, when part of the difficulty lies in target specification itself [4,7].

DSM-5 attracted precisely this kind of criticism. Several authors argued that its revisions did not solve the deeper problem of diagnostic validity and, in some areas, risked preserving or worsening clinically important misclassification. The debate over depression with mixed features is instructive. Koukopoulos, Sani, and Ghaemi argued that DSM-5 criteria for mixed features failed to capture the clinical reality of mixed depression, particularly by excluding symptoms such as psychomotor agitation, irritability, inner tension, and affective lability that had long been central to clinical descriptions of mixed depressive states [8,9,10,11]. It raised the more serious possibility that an operationally tidy specifier could obscure a clinically meaningful condition and contribute to inappropriate treatment decisions. Ghaemi’s broader criticism of DSM-5 made the same point at the level of psychiatric nosology: reliability, committee consensus, and descriptive convenience do not by themselves establish diagnostic validity.

These DSM-5 criticisms matter for the present argument because they show that the problem is not limited to biological research targets. A diagnostic construct may be administratively useful and formally operationalized while still failing to map onto a clinically meaningful illness structure. Conversely, a clinically recognizable presentation may be poorly represented by official criteria. The issue is therefore not simply whether psychiatry should use categories or dimensions, but whether any classificatory construct—categorical, dimensional, or hybrid—has earned the level of validity being claimed for it.

The empirical consequences are now difficult to ignore. Within diagnoses, heterogeneity is marked [4,12]. Patients meeting criteria for major depression, schizophrenia, bipolar disorder or obsessive–compulsive disorder may differ substantially in onset, symptom pattern, cognitive profile, functional burden, treatment response and trajectory [12,13,14]. Across diagnoses, comorbidity is not peripheral noise but a central feature of the clinical picture [4,15,16,17]. Longitudinal data make the point more clearly than cross-sectional surveys. In the Dunedin Study, mental disorders across four decades showed extensive comorbidity, persistence and heterotypic continuity [18]. What appears cross-sectionally as several disorders often looks longitudinally like shifting expressions of broader liabilities [17,18].

This helps explain why diagnosis-bound biomarker discovery has so often disappointed [19,20,21]. Psychiatry has learned a great deal from genetics, imaging and molecular work. The problem is not the absence of biology but the weakness of the syndromic target [19,20,22,23]. The recent *World Psychiatry* review of candidate biomarkers was appropriately sober on this point: despite a vast literature, psychiatry still lacks biomarkers robust enough to guide most routine diagnostic decisions, and part of the shortfall reflects the complexity and heterogeneity of the phenotypes themselves [19]. In practice, biology has often been asked to hit a target that was too syndromically bounded and too internally diverse to be biologically tractable [19,20].

Kendler’s broader argument follows from this. Psychiatric disorders are unlikely to yield a simple etiological taxonomy analogous to classical infectious disease models [7,24,25]. Causation is probabilistic, distributed across levels, developmentally contingent and environmentally modulated [7,25]. That is not an argument against diagnosis. It is an argument against treating DSM and ICD syndromes as final biological entities [4]. The more plausible biological targets are cross-cutting liabilities, dimensions of expression, and trajectories of progression rather than syndromic categories considered in isolation [4,26,27,28].

On this basis, the present review was guided by three questions. First, how should descriptive or predictive psychiatric dimensions be distinguished from candidate disease axes? Second, what evidential thresholds should be required before a dimensional or transdiagnostic construct is granted provisional disease-axis status? Third, how should major contemporary frameworks, including Research Domain Criteria (RDoC), Hierarchical Taxonomy of Psychopathology (HiTOP), the p factor, cross-disorder genomic models, clinical staging approaches, and data-driven subtyping, be interpreted when judged against those thresholds? These questions structure the conceptual synthesis that follows.

## 2. Conceptual Analysis Strategy

This article was designed as a conceptual synthesis aimed at framework development, not as a systematic review, scoping review, or meta-analysis. Its purpose was to clarify the evidential conditions under which dimensional or transdiagnostic psychiatric constructs may warrant provisional interpretation as candidate disease axes. The central task was therefore epistemological and methodological: to distinguish descriptive structure, predictive utility, and disease-level biological validity, and to specify the inferential safeguards required before a psychiatric construct is promoted to disease-axis status.

The literature search was used to support this conceptual analysis rather than to generate an exhaustive evidence inventory. We conducted targeted and purposive searches in PubMed/MEDLINE, Scopus, Web of Science, and Google Scholar from database inception to 15 April 2026. Searches combined terms related to psychiatric classification, dimensional psychopathology, transdiagnostic models, and biological validation, including “psychiatric nosology”, “DSM”, “ICD”, “biological validity”, “dimensional psychopathology”, “transdiagnostic”, “RDoC”, “HiTOP”, “p factor”, “psychiatric genetics”, “clinical staging”, “biotypes”, “machine learning”, and “biomarkers”. Additional sources were identified through backward and forward citation tracking from key articles, reviews, and consensus papers.

Source selection was purposive and iterative. We prioritized publications that were conceptually central, methodologically influential, or directly relevant to psychiatric construct validation. These included work on limitations of DSM/ICD categories as biological targets; major dimensional and transdiagnostic frameworks; cross-disorder genomic and neurobiological evidence; developmental and staging models; data-driven subtyping; biomarker validity; and philosophical or methodological critiques of diagnostic validity, utility, and reification. Sources were not selected to estimate pooled effects, compare interventions, or provide a graded summary of all available evidence.

Accordingly, the search strategy should not be interpreted as a systematic, scoping, or semi-systematic review methodology. We did not conduct preregistration, dual independent screening, PRISMA flow-charting, risk-of-bias assessment, formal quality scoring, or quantitative synthesis. Instead, the identified literature was used to inform a conceptual appraisal of how psychiatric constructs move, or fail to move, from descriptive or predictive usefulness toward stronger claims of disease-level biological validity.

The synthesis proceeded by separating three levels of inference that are frequently conflated in psychiatric research: descriptive structure, predictive utility, and disease-level biological validity. On this basis, we developed a cumulative framework for assessing when a psychiatric construct may move from useful latent structure toward provisional candidate disease-axis status.

## 3. Why Psychiatry Turned to Dimensions

Psychiatry turned toward dimensions not because categories ceased to be clinically useful but because they proved weak as final biological targets [7,9,29]. Symptom patterns, comorbidity and longitudinal course did not behave as a set of discretely bounded disorders should [28,29,30]. The dimensional turn was therefore not a matter of theoretical fashion. It was an empirically driven response to repeated mismatches between syndromic description and the observed structure of psychopathology [28,30].

One response was RDoC. By shifting the unit of study from consensus diagnoses to dimensional systems of function, RDoC reopened questions that DSM-era research had too often bracketed [7,9,27]. Its real contribution was not to solve psychiatric nosology, but to loosen the field’s dependence on diagnosis as the compulsory starting point for biological inquiry [9,27,31]. It gave investigators permission to study threat, reward, cognition and arousal across conventional boundaries [27,32,33]. That was a major advance. But it addressed the problem of the research unit, not the problem of the disease unit [7,33]. A tractable functional construct is not yet a disease axis [33].

A second response came from the quantitative mapping of psychopathology itself. HiTOP and related hierarchical models showed with increasing consistency that symptoms and disorders organize into broader spectra such as internalizing, externalizing and thought disorder [7,28,29,34,35]. This area of the literature mattered because it began from the observed structure of psychopathology rather than from prior diagnostic committees [28,29,35]. Its limitation is equally important. A hierarchy of symptoms is not the same thing as a hierarchy of diseases [7,28,35]. HiTOP estimates the structure of psychopathology; it does not by itself decide which dimensions deserve biological standing [7,35].

The general psychopathology factor (p factor) sharpened the problem further. Caspi and colleagues showed that internalizing, externalizing and thought disorder could themselves be summarized by a general psychopathology factor associated with persistence, impairment, adverse developmental histories and broad burden [26,36,37,38,39,40]. That mattered because it made clear that comorbidity was not peripheral noise and because it helped explain why biological correlates of disorder so often appear nonspecific [26,38,40]. But the p factor did not resolve the status of general psychopathology [40,41]. It showed that broad liability can be modeled. It did not show that such liability should be treated as a primary disease axis [40,41]. Dimensionality became difficult to deny. Biological privileging did not [30,40,41].

Genetics gave the dimensional shift its strongest external support. Cross-disorder studies progressively undermined the assumption that major psychiatric disorders are etiologically discrete [42,43,44,45]. Common variant sharing across psychiatric conditions, pleiotropic loci and multivariate genomic models all pointed in the same direction [42,44,45,46,47]. Category-first biology had been an inadequate starting point [42,45,46]. But shared liability is not the same thing as nosological collapse. The bipolar disorder-schizophrenia dissection remains instructive precisely because it shows overlap and differentiation at once [42,43,46,47]. What emerges is not a set of sealed categories, but neither is it one featureless continuum. It is a landscape of partially overlapping liabilities [42,45,46,47].

Developmental psychiatry added another form of pressure that static nosology could not absorb easily. Clinical staging and transdiagnostic youth frameworks grew from the recognition that early psychopathology is often pluripotent, fluid and only later more syndromically differentiated [48,49]. The key developmental question is not simply whether a construct is stable over time, but whether it shows lawful transformation across age and stage [48,50,51]. This is why developmental coherence belongs near the center of any account of biological validity [48,51]. A psychiatric construct that looks persuasive cross-sectionally but has no intelligible developmental life history has probably not yet reached the level of disease architecture [48,50,51].

Taken together, these developments did not simply show that psychopathology is dimensional—they showed that psychiatry had changed its preferred units of description without yet establishing disciplined rules for deciding which of those units deserve disease-level standing [7,27,28,30,35]. It moved into dimensions without disciplined rules of ontological promotion. What changed, then, was not only the preferred unit of description but also the burden of proof required before description could be treated as disease architecture.

## 4. From Latent Structure to Disease Axes

What psychiatry currently lacks is not more latent structure but a disciplined rule for deciding which constructs deserve disease-level standing. The field has accumulated dimensions, spectra, subtypes and signatures faster than it has developed rules for deciding what kind of claim each of them can bear [7,13,52]. As a result, descriptive constructs, strategically useful constructs, and candidate disease axes are too often treated as though they made the same claim on reality. They do not, but some summarize covariance [28]. Some improve prediction or stratification [29,30]. Only a smaller subset deserves promotion to disease-level standing [31,53]. The central question is not whether a construct is dimensional or biologically informed. It is whether the evidence is strong enough to treat it as part of disease architecture rather than as a useful summary of data [33,54].

In this article, the terms category and dimension are used in their contemporary nosological and psychometric sense, not in the classical Aristotelian sense of categories as the most general forms of predication. In that older philosophical usage, dimensions may indeed be considered categories. That observation is correct, but it does not dissolve the psychiatric problem addressed here. In DSM/ICD practice, a categorical construct assigns a patient to a bounded class, usually through threshold criteria, whereas a dimensional construct orders patients along a graded continuum of liability, symptom expression, function, severity, or progression without assuming an intrinsic natural cut-point. Thus, the relevant distinction is not metaphysical category versus non-category, but bounded classification versus graded measurement and inference.

For the purposes of this review, a psychiatric dimension is defined operationally as an ordered quantitative or quasi-quantitative construct on which individuals can differ by degree, derived from clinical, behavioral, biological, or longitudinal observations, and interpretable only within stated measurement and validation rules. This definition deliberately does not imply biological reality. A dimension may be descriptive, prognostically useful, or mechanistically suggestive without being a disease axis. The disease-axis claim requires the additional evidential thresholds set out below.

A statistical dimension summarizes covariance. It may be stable, predictive and mathematically elegant, but reproducible covariance is evidence of structure, not yet of ontology [37,55]. Factors may reflect shared causes, consequences, measurement artifacts, ascertainment biases or generic severity [13,56]. Replication narrows these possibilities; it does not resolve them [28]. Factor stability alone is not biological validation [54,55]. The same slippage recurs in more recent forms: reproducible clusters are too easily called subtypes, classifiers are treated as mechanisms, and embeddings are mistaken for architecture [26,30,57,58]. Predictive success authenticates performance, not the nature of the construct being predicted [29,53].

A disease axis should be defined more narrowly. It is not just a dimension that organizes symptoms well. It should also track illness across time, connect to evidence beyond symptom geometry, and matter clinically [26,31,33]. Such an axis need not correspond to a single pathway, lesion or biomarker [52]. Psychiatry is too causally distributed for that expectation to be realistic [55,59]. But it should function as a genuine organizing dimension of illness rather than as a convenient compression of data.

The term is not meant to rename any transdiagnostic construct that performs well statistically or biologically. A validator-rich nosological construct may gain external support without yet organizing illness across time and clinical consequence. A transdiagnostic liability construct may capture broad vulnerability without yet distinguishing how that vulnerability is expressed, develops, or shapes prognosis. A mechanistically constrained phenotype may narrow causal interpretation without yet structuring clinical course. A stratification variable may improve prediction, monitoring, or trial enrichment without carrying stronger implications for disease architecture. A disease axis, as used here, is meant more narrowly: it refers to a construct that begins to organize liability, expression, or progression of illness in a clinically consequential and developmentally intelligible way.

This distinction matters because neither broadness nor narrowness guarantees validity [4]. A general severity factor may capture much of the psychiatric burden without identifying a specific pathophysiological axis [5,37]. Conversely, a narrow experimental construct may appear mechanistic while tracking an ancillary process or task artifact rather than disease organization [5,58]. The crucial issue is not whether a construct is dimensional, neural, computational or transdiagnostic. It is whether it survives the tests required for promotion to disease-axis status [31,33,59].

A useful demarcation follows. Descriptive constructs organize the architecture of symptoms and related observations [5,13]. Strategically useful constructs improve prognosis, stratification, staging or trial design even when their mechanistic status remains limited [29,49,60]. Candidate disease axes warrant stronger biological standing because they organize liability, expression or progression in a way that survives cumulative validation across levels, time and consequence [26,31,53]. The field’s recurrent error has been premature ontological upgrading: treating descriptive or strategically useful constructs as though they had already earned disease-level status [7,30,54]. The central problem is therefore not dimensionality itself, but the absence of disciplined rules for deciding which dimensions are allowed to count as disease architecture [7,52]. For that reason, the concept of a disease axis is intended to mark an epistemic and organizational distinction, not merely a rhetorical upgrade in prestige. It refers to a construct that begins to organize the architecture of illness itself (its liabilities, forms of expression, or trajectories of progression) rather than serving only as a validator-rich correlate, a useful risk marker, or a mechanism-facing phenotype.

The difficulty is not that psychiatry lacks validators. It is that the field has repeatedly allowed single validators to carry more ontological weight than they can bear. Heritability, neural correlation, predictive performance, clustering, and experimental tractability may each support a construct, sometimes strongly, yet none is sufficient on its own to justify disease-level standing. The resulting pattern has been recurrent over-promotion: constructs that proved descriptively useful, biologically suggestive, or statistically impressive were too readily treated as if they had already identified disease architecture. The task, therefore, is not to add one more validator to the list, but to specify the minimum safeguards required before stronger ontological claims are made.

## 5. From Validation to Ontological Promotion: An Eight-Domain Hierarchical Framework for Candidate Disease Axes

To avoid ambiguity, the framework is organized hierarchically. We use domain as the general term for the eight evidential requirements discussed below. Four domains function as core gatekeepers: replication across cohorts and methods, developmental coherence, prognostic increment beyond diagnosis and nonspecific severity, and discriminability from nonspecific severity. These are treated as minimal requirements because failure in any of these areas commonly reflects one of the major routes to false ontological promotion in psychiatry.

The remaining four domains are supporting or disciplining domains: cross-level convergence, mechanistic constraint, clinical leverage, and falsifiability with explicit boundary conditions. These domains do not replace the core gatekeepers. Rather, they strengthen or constrain candidate disease-axis claims once the minimum gatekeeping requirements have been met. In this sense, the framework is not a flat checklist, but a hierarchical decision structure: core gatekeepers determine whether promotion is even defensible, whereas supporting and disciplining domains determine how strong, clinically meaningful, and falsifiable that promotion claim is.

Existing validation logics remain necessary, but they are no longer enough [4,5,7]. Classical discussions of diagnostic validity, utility, staging, transdiagnosticity and mechanism identify important virtues of psychiatric constructs, but they do not by themselves resolve the problem of ontological rank [5]. The issue is no longer whether a construct is useful, but whether it satisfies the promotion criteria required for candidate disease-axis status [61,62,63]. What psychiatry now lacks is not another validator in isolation, but a rule for deciding when convergent evidence is strong enough to justify promotion [24,64]. No single signal should be enough on its own.

The need for a cumulative filter does not arise from a preference for integrative models as such. It arises from repeated failure of single validators to bear ontological weight in psychiatry. Heritability does not identify a disease unit by itself. Neural correlation does not identify a disease unit by itself. Longitudinal prediction does not identify a disease unit by itself. Nor do clustering solutions, machine-learning classifiers, or experimentally tractable constructs. Each of these may contribute important evidence, but each has also repeatedly licensed claims that later proved too broad, too unstable, too severity-bound, or too weakly anchored to the illness course. The present framework is therefore intended not as an exhaustive theory of validity, but as a conservative safeguard against premature ontological promotion.

The framework should therefore not be read as a discovery procedure for psychiatric reality. Its purpose is narrower and more disciplinary: to regulate how far available evidence can reasonably support stronger ontological claims. It is meant to constrain inference, not to replace judgment, and not to function as an algorithm for deciding what psychiatric disease architecture ultimately is.

In psychiatry, biological standing must be earned cumulatively, not inferred from any single validator [19,65]. Heritability is insufficient [66,67,68]. Neural correlation is insufficient [69,70]. Longitudinal prediction is insufficient [71]. Even treatment association is insufficient [19,69]. A putative disease axis deserves promotion only if it survives a cumulative validation filter. For that filter to be more than a statement of principle, it must also be usable [20]. Each criterion should therefore be interpreted operationally rather than rhetorically. In practice, that means asking what would count as enough evidence, what would count as limited support, and what should count as failure [69]. The question is not whether a construct shows some signal somewhere. The question is whether independent investigators could apply comparable standards and reach broadly similar conclusions about it [20].

In practice, this means framing each criterion as a threshold question: what pattern of evidence counts in favor of promotion, what counts against it, and what still leaves the construct provisional [19].

Each criterion should be judged at one of three levels: met, provisional/partial, or failed. A criterion is met when the relevant evidence is replicated and materially constrains inference; provisional when a signal exists but remains limited in portability, consistency, or practical consequence; and failed when the construct does not yet show the required evidence or loses support after appropriate controls. To avoid terminological ambiguity, Table 1 lists the eight evidential domains using the same wording as Section 5 subheadings and identifies whether each domain functions as a core gatekeeper or as a supporting/disciplining domain.

Table 2 then provides operational thresholds for judging the four core gatekeepers as met, provisional, or failed. They are best understood as judgments about the evidential defensibility of promotion claims under current knowledge. A construct may satisfy several such conditions and still remain an imperfect, revisable model of illness organization rather than a final representation of disease reality.

The criteria are not all intended to play the same evidential role. Some act primarily as safeguards against recurrent inferential errors; others strengthen confidence that a construct captures more than symptom geometry; still others prevent immunization against disconfirmation. This distinction matters because the framework is meant to discipline promotion, not merely to accumulate favorable signals.

The eight evidential domains together define the framework, but only four function as core gatekeepers. No psychiatric construct should be promoted from latent structure to candidate disease architecture on the basis of any single validator or any single level of analysis [24,63,64]. Four of these tests function as core gatekeepers because each addresses a recurrent route to false elevation in psychiatric nosology.

### 5.1. Cross-Level Convergence

The first test is structured convergence across levels of analysis [61,62,63]. A biologically credible axis should recur in a non-trivial pattern across more than one level: symptom expression, cognition, behavior, physiology, genomics, molecular findings, neural systems and longitudinal course [24,64]. The operative standard is not association with many things, which broad severity constructs often achieve with ease, but patterned alignment that makes the construct more intelligible rather than more diffuse [61,62].

Structured convergence should not be confused with the mere accumulation of correlates across levels [62,63]. The relevant question is whether those associations form an interpretable pattern that narrows plausible models of liability, expression or progression, rather than simply surrounding a clinically broad construct with a biological signal [24,64].

Genetic sharing across disorders, multivariate genomic factors, shared transcriptomic perturbations, and partially convergent neuroimaging patterns may all count as forms of cross-level evidence [61,62,63,64,65,66,67,68,70,71,72]. But no single level should dominate adjudication [19,24]. A poorly anchored biomarker is not rescued by its biological appearance [19,20,69]. Conversely, a symptom spectrum with replicated developmental and prognostic structure may carry more biological weight than a neural signature that lacks durable phenotypic anchoring [61,62,71].

### 5.2. Replication Across Cohorts, Ascertainment and Method

The second test is replication under conditions that matter [19,69]. A disease axis should recur across independent cohorts, ascertainment strategies, analytic pipelines, instruments, and, where possible, ancestries and health-care settings [20]. Replication within closely similar convenience samples is a start, not a finish [73,74,75]. Without portability, the construct may reflect local structure rather than disease structure [74,76].

For present purposes, replication should mean more than repeated appearance in closely related datasets. The stronger standard is recurrence across independent cohorts with different ascertainment frames or analytic pipelines, such that the construct remains recognizable despite changes in instrument, sample composition or method [69]. A finding that depends heavily on one pipeline, one modality or one narrow recruitment strategy should remain provisional, however striking it appears in its original report [20,74].

This criterion is especially unforgiving in neuroimaging and machine learning [69,70,74,75]. Small samples and flexible pipelines have repeatedly produced impressive but unstable results [72,74]. Genetic findings raise a related issue: a pattern that holds only in heavily European-ancestry datasets cannot automatically be treated as general disease architecture [72,73]. Measurement invariance and population portability are not auxiliary concerns [76]. They are part of validity itself [69,76].

Operationally, this criterion should be treated as met when the construct recurs across independent cohorts, measurement strategies, and analytic pipelines without major changes in its interpretive meaning. It should be treated as provisional when replication is limited to closely related datasets, similar instruments, or a narrow ascertainment frame. It should be treated as failed when the construct depends heavily on one pipeline, one modality, or one restricted sample, or when it changes substantially under modest methodological perturbation.

### 5.3. Developmental Coherence

The third test is developmental coherence. Psychiatric validity is inherently temporal. A biologically meaningful axis should show an intelligible developmental life history: antecedents, age-sensitive expression, persistence or transformation, and relations to progression, remission or stage transition. Stability alone is not enough. A convincing axis may change across development, but it should do so lawfully.

This is one reason broad transdiagnostic spectra can be more credible than isolated cross-sectional clusters [61]. Internalizing liability shows temperamental antecedents and age-linked expression. The p factor is compelling partly because it summarizes persistence and heterotypic continuity as well as contemporaneous comorbidity. Staging models sharpen the point further by showing that clinically important structures may lie in trajectories, not only in profiles. Psychiatric disease axes are likely to be trajectory-bearing, not merely cross-sectional descriptors.

This criterion should be treated as met when the construct shows a recognizable developmental life history, including antecedents, age-sensitive expression, and lawful persistence, transformation, or progression. It should be treated as provisional when longitudinal evidence exists but is sparse, short-term, or limited to cross-sectional age gradients. It should be treated as failed when the construct has no intelligible developmental profile or when its apparent structure does not survive longitudinal examination.

### 5.4. Prognostic Increment Beyond Diagnosis and Nonspecific Severity

The fourth test is prognostic increment. A dimension earns scientific standing when it improves the prediction of outcomes that matter to patients and clinicians: persistence, relapse, chronic disability, suicidality, functional decline, hospitalization, treatment resistance or conversion. In psychiatry, a prognostic signal is often a stronger validator than cross-sectional biological correlation because prognosis forces the construct to show that it tracks illness course rather than only symptom geometry [71].

Prognostic increment should not be claimed on the basis of statistical significance alone. The relevant question is whether the construct reproducibly improves prediction of clinically consequential outcomes beyond diagnosis and nonspecific severity, and whether that improvement is large enough to alter stratification, follow-up intensity, preventive targeting or trial design [71].

The threshold is practical rather than merely statistical. If a construct improves model fit yet does not change any consequential prediction beyond diagnosis and crude severity, it may still be descriptively useful, but it has not yet crossed the threshold required for ontological promotion [23,73]. This is why some broad spectra deserve to be taken seriously: they are not just statistically tidy; they predict recurrence, burden and impairment. A dimension that reorganizes covariance but adds nothing to prognosis should remain provisional [71].

This criterion should be treated as met when the construct reproducibly improves prediction of clinically consequential outcomes beyond diagnosis and nonspecific severity and when that improvement is large enough to alter stratification, monitoring, prevention, or trial design. It should be treated as provisional when predictive gain is statistically detectable but small, inconsistent, or clinically inert. It should be treated as failed when the construct adds no meaningful prognostic information beyond existing diagnostic and severity-based models.

A practical contrast helps clarify the point. A broad internalizing spectrum may justify a stronger interest because it does more than summarize symptoms cross-sectionally: it shows recognizable developmental antecedents, predicts persistence and impairment, and retains greater interpretive value than a mere global burden score. By contrast, a general psychopathology factor may capture overall severity and long-term burden very well, yet still remain less discriminating as a candidate disease axis if much of its apparent depth is lost after explicit modeling of nonspecific severity.

### 5.5. Mechanistic Constraint

The fifth test is a mechanistic constraint [24,63,64]. Biological validity does not require a complete causal account, but it does require that the construct constrain plausible causal models [63,64]. The point is not to produce a full mechanism, but to narrow the search space and permit more specific inferences than the generic claim that “brain dysfunction is involved.” [24,63].

Mechanistic constraint should not be inferred from biological association alone [63,64]. The relevant standard is whether the construct excludes at least some otherwise plausible interpretations and narrows the range of viable causal models [22,63]. A dimension that can be linked post hoc to many different biological stories remains suggestive. A dimension that consistently points toward a smaller and more coherent set of processes has begun to exert genuine mechanistic constraint [63,64].

Genetics has contributed here more than most other levels [66,72,73]. Cross-disorder loci enriched for neurodevelopmental biology, broad genetic factors with distinguishable functional profiles, and transcriptomic overlap paralleling polygenic overlap do not amount to a full explanation [66,72,73]. But they do show that some dimensions are constrained by identifiable classes of biological process [66,72]. A factor that cannot be connected to any plausible causal architecture except by post hoc narrative remains, at best, an efficient statistical summary [63].

The same logic applies in the opposite direction. A construct may appear biologically attractive because it narrows causal interpretation or clusters patients into plausible subgroups, yet still fails to organize illness in a clinically durable way. This is why some biotypes remain methodologically interesting without yet becoming disease axes: they may constrain mechanism more than they stabilize prognosis, development, or portability across cohorts.

### 5.6. Discriminability from Nonspecific Severity

The sixth test is discriminant specificity. A disease axis must be distinguishable from generic distress, burden, chronicity or referral severity. This is one of the field’s most neglected problems. Broad constructs are attractive because they absorb messy covariance, but the ease with which they capture everything can also mean that they differentiate little.

The p factor embodies this tension. It clearly indexes global liability and burden. But whether it captures a substantive shared pathophysiology, generalized severity, cumulative adversity, chronicity, or some mixture of these remains unresolved. Comparative evidence suggests that middle-level spectra may carry a more stable and interpretable signal than a universal factor [61]. The same logic applies elsewhere. Shared genetic liability does not make the distinction between psychotic, compulsive and internalizing liabilities biologically trivial [66,72,73]. Overlap must be demonstrated; nonspecificity must not be smuggled in as depth.

This criterion should be treated as met when the construct remains interpretable after adjustment for generic burden, chronicity, and referral severity, and when it retains differentiating value beyond global impairment. It should be treated as provisional when some specificity is suggested, but residual confounding by severity remains plausible. It should be treated as failed when the construct largely collapses into general distress, cumulative burden, or chronicity once those factors are modeled explicitly.

### 5.7. Clinical Leverage

The seventh test is clinical leverage. A dimension that changes no meaningful clinical inference remains provisional, however elegant its statistics. Clinical leverage does not require immediate bedside implementation, nor should psychiatry demand instant utility as the price of scientific seriousness. But a putative disease axis should at least improve formulation, stratification, prognosis, prevention, monitoring, trial design or treatment selection beyond diagnosis plus crude severity.

Clinical leverage should not be reserved for immediate treatment matching, but neither should it be reduced to general clinical interest. A construct has clinical leverage when it changes formulation, risk estimation, staging, monitoring, enrichment or prevention in ways that diagnosis alone would not support. The threshold is crossed when the construct changes what a clinician, service or trialist would reasonably do next.

This is where many fashionable constructs fail [70,74,75]. Dimensional profiles can improve formulation and communication, as HiTOP proponents have argued [61]. Staging adds clinically usable information that diagnosis alone misses. By contrast, many biotypes and neural signatures remain too unstable, context-bound or opaque to alter care defensibly [70,74,75]. The translational bar should not be impossibly high, but it should be real.

### 5.8. Falsifiability and Boundary Conditions

The eighth test is falsifiability. Psychiatric constructs are too often protected by elasticity. If a factor replicates, it is declared robust; if it does not, it is called context-sensitive. If it lacks specificity, it is praised as transdiagnostic; if it lacks clinical relevance, it is described as upstream [63]. This is how constructs become immunized against evidence.

A credible disease axis should therefore come with stated boundary conditions [19,63]. What findings would count against it? Failure to replicate across ascertainment strategies; disappearance after adjustment for severity or method variance; absence of developmental continuity; lack of prognostic increment; inability to travel across ancestries or settings; or failure to identify any coherent multilevel pattern should all weaken the claim [19,63,71,74,76]. The field should become much harder to impress [19,63].

A construct should remain provisional when it shows promising structure but lacks one or more of the following: robust replication across methods and cohorts, longitudinal or developmental coherence, discriminability from generic severity, or evidence that it changes clinical or mechanistic inference [19,63,71,74,76]. Candidate disease-axis status becomes more defensible when these thresholds are met jointly rather than serially or selectively [19,63]. The point is not to eliminate judgment, but to discipline it.

Taken together, these tests are meant to do more than reward promising constructs. They are meant to guard against the specific ways psychiatric constructs are repeatedly over-read: as local findings mistaken for general structure, as cross-sectional regularities mistaken for illness architecture, as burden variables mistaken for biological depth, and as statistically successful models mistaken for clinically meaningful disease organization. The threshold structure of the framework follows from these recurrent failure modes rather than from a preference for comprehensiveness alone.

### 5.9. Hierarchy of Evidential Roles Within the Framework

Not all criteria in the framework play the same evidential role, because not all are designed to answer the same inferential risk. Some are best understood as core gatekeepers against premature promotion, whereas others provide constraining or confidence-increasing evidence once those safeguards have been met [19,63]. Four domains are treated here as core gatekeepers rather than merely desirable validators: replication across cohorts and methods, developmental coherence, discriminability from nonspecific severity, and prognostic increment beyond diagnosis and crude burden. They are privileged not because they exhaust the problem of validity, but because each addresses a distinct and recurrent route to false elevation in psychiatric nosology. Replication guards against local or pipeline-dependent structure. Developmental coherence guards against reifying cross-sectional order as disease architecture. Discriminability from nonspecific severity guards against mistaking broad burden for biologically meaningful organization. Prognostic increment guards against promoting statistically tidy constructs that do not change clinically consequential inference [19,63,69,71,77].

For the same reason, the framework should not be interpreted as conferring ontological rank in any strong metaphysical sense. It does not determine what a construct really is. It disciplines what the field is entitled to claim about that construct, given the recurrent tendency to mistake descriptive order, predictive success, or biological signal for disease architecture itself.

The point is therefore not that other criteria matter less in the abstract. Cross-level convergence, mechanistic constraint, and clinical leverage remain highly relevant. But they do not by themselves protect against the most common forms of over-interpretation in psychiatry. For that reason, they are better treated as strengthening domains than as substitutes for the core gatekeepers. Falsifiability and explicit boundary conditions serve a different function again: they do not provide positive support on their own, but they prevent the construct from being protected by interpretive elasticity.

Other criteria play a supporting or constraining role [24,63,64]. Cross-level convergence and mechanistic constraint increase confidence that the construct captures more than symptom geometry, while clinical leverage strengthens the case that the construct reorganizes clinically meaningful inference [19,24,63,64]. Falsifiability and explicit boundary conditions operate differently again: they do not by themselves establish disease-axis status, but their absence weakens the construct by leaving it insufficiently exposed to disconfirmation [67].

The framework also allows for different validation profiles across constructs [19,63]. Not every candidate disease axis should be expected to satisfy every supporting criterion in the same way or to the same degree [19,63]. Some constructs may derive their strongest claim from developmental and prognostic coherence, others from multilevel convergence or dynamic relevance to progression [24,63,64]. What should remain invariant is the minimum threshold for promotion: the construct must satisfy the core requirements and show at least one additional source of constraining evidence [19,63]. The point is not that every construct must look the same. The point is that promotion should follow explicit thresholds rather than preference or fashion [19,63].

Certain findings should count not merely as missing support, but as reasons to stop promotion [19,63]. These include failure to replicate across ascertainment strategies or analytic pipelines, disappearance of the construct after adjustment for generic severity or method variance, lack of developmental continuity, absence of meaningful prognostic increment, and instability across populations or settings [19,63,71,74]. Such findings need not imply that a construct is useless, but they should block its advancement to candidate disease-axis status [19,63].

### 5.10. Minimal Decision Rule

We therefore treat stronger claims to candidate disease-axis status as defensible only when a construct survives all four core gatekeepers, shows at least one additional source of constraining evidence, and is not weakened by a disqualifying condition such as instability across methods, collapse into nonspecific severity, or loss of developmental or prognostic coherence. This is intended as a conservative framework for disciplining ontological promotion claims, not as a formal algorithm for identifying psychiatric reality. Its purpose is not to confer authority on promising constructs but to reduce false ontological promotion (Table 1). Any subsequent application of the framework, including the comparative judgments shown in Table 3, should therefore be read as a disciplined interpretive exercise under stated thresholds rather than as the output of a formal scoring algorithm.

A construct should remain provisional when it shows a meaningful signal in some domains but does not yet survive the core gatekeepers jointly. In such cases, the construct may remain descriptively useful, strategically valuable, or mechanistically suggestive, but stronger disease-axis claims should remain deferred [19,24,63,64,71]. It should not be promoted when any two core criteria fail, when discriminability from nonspecific severity fails, or when the construct proves unstable across cohorts, methods, or populations [19,63,74,76].

The main value of the framework lies not in confirming easy cases, but in adjudicating difficult ones. Its point is to distinguish between constructs that are broadly predictive but nonspecific, constructs that are mechanistically informative but nosologically incomplete, and constructs that combine replication, developmental coherence, prognostic increment, and sufficient specificity to justify provisional promotion.

The framework is intentionally conservative for that reason. In a field repeatedly shaped by premature reification, the more important task is not to identify winners quickly, but to make promotion harder unless the construct survives the main historical sources of inferential error.

**Figure 1:**
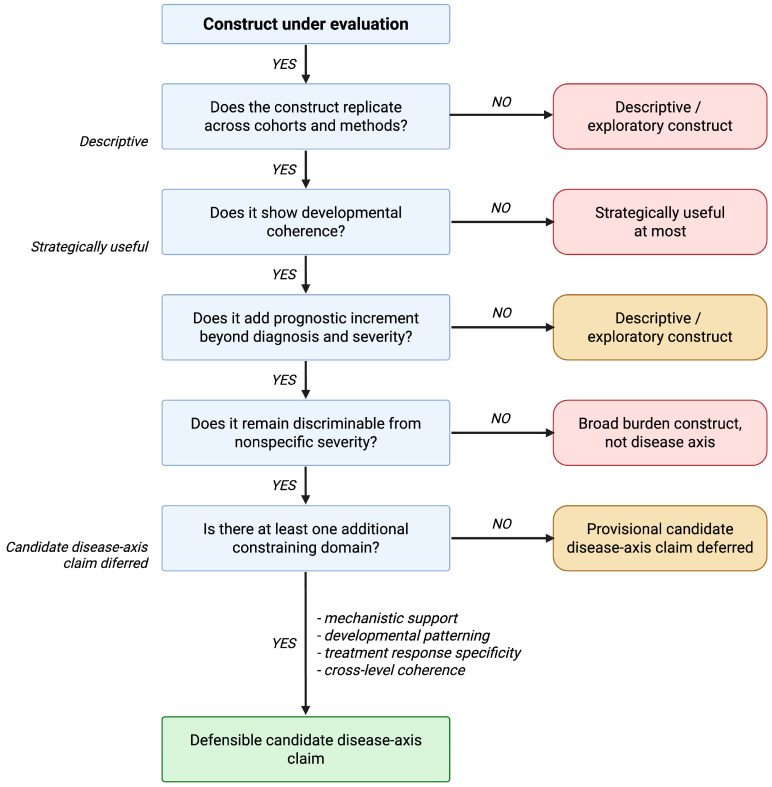
Decision pathway for disciplined ontological promotion from construct to candidate disease-axis claim.

In effect, the framework asks not whether a construct is interesting, but whether the field has earned the right to speak about it as organizing illness rather than merely summarizing data. Figure 1 translates this cumulative filter into a staged decision pathway for disciplined ontological promotion, showing how constructs should move from descriptive or strategically useful status toward a defensible candidate disease-axis claim only after passing explicit inferential thresholds.

## 6. How the Framework Should Be Used

The framework is intended as a threshold tool rather than a rhetorical ideal [19,63]. Its use should remain inferential rather than constitutive. That is, the framework is not designed to declare what psychiatric constructs are in themselves, but to discipline how confidently they may be interpreted as organizing dimensions of illness rather than as descriptive summaries, strategic tools, or provisional models. In practice, its use involves five steps: locating the construct at the correct epistemic level; assessing evidence across the four core gatekeepers; requiring at least one supporting/disciplining domain; stating boundary conditions explicitly; and assigning a provisional status of descriptive, strategically useful, candidate disease axis, or unsupported [19,63]. Table 3 applies this logic to major current constructs.

The application shown in Table 3 is intended as a structured illustrative exercise rather than as a formal rating procedure. The judgments reported there do not arise from quantitative synthesis or a pre-registered scoring protocol. They reflect reasoned comparative appraisal under the evidential thresholds defined in the framework: whether a construct shows replicated support for the four core gatekeepers, whether at least one additional constraining domain is credibly present, and whether any disqualifying condition substantially weakens the claim. The purpose of the table is therefore not to issue final verdicts, but to make the grounds of comparison explicit and contestable.

### 6.1. Worked Application: Internalizing Spectrum Versus P Factor

A brief worked application may clarify how the framework should be used. Table 4 applies the four core gatekeepers to two familiar constructs: the HiTOP internalizing spectrum and the general psychopathology factor. These examples are useful because both constructs are empirically important, but they make different claims on disease-level validity. The internalizing spectrum is a middle-level construct that captures patterned covariation among depressive, anxiety, fear, and related syndromes. The p factor is a higher-order construct that summarizes broad liability and burden across psychopathology.

In this application, the HiTOP internalizing spectrum performs relatively well because it has been replicated across hierarchical models, shows recognizable developmental antecedents and age-linked expression, and carries prognostic information regarding persistence, recurrence, and impairment. Its main limitation is discriminability: it must remain separable from generic distress, chronicity, and total symptom burden before stronger disease-axis claims become defensible. The p factor shows a different profile. It is robust as a descriptive summary of broad psychopathology and is often associated with impairment, persistence, and adverse developmental histories. However, its disease-axis status remains less secure because its breadth is also its main liability: the construct may capture global severity or cumulative burden more than a specific organizing dimension of illness. Thus, the framework does not treat the p factor as unimportant; it treats it as insufficiently discriminating for disease-axis promotion under current evidence.

### 6.2. Testable Implications of the Framework

A useful promotion framework should not merely classify constructs retrospectively; it should generate falsifiable expectations [19,63]. Under the present framework, candidate disease axes should remain recognizable across instruments, cohorts, ascertainment frames and analytic pipelines rather than collapsing under modest changes in method [19,63,74,76]. Broad liability constructs such as the p factor should lose much of their apparent biological depth if they are driven primarily by nonspecific burden, chronicity or referral severity [61]. Proposed biotypes should not be granted disease-level standing unless they outperform simpler dimensional models in replication, prognostic increment and portability across samples [70,74,75]. Dynamic staging constructs should justify their standing by improving prediction of progression, persistence or transition beyond static symptom profiles alone [71]. A framework that cannot generate such differential expectations would remain classificatory rhetoric rather than a genuine tool for scientific adjudication [19,63]. If the framework generates differential predictions, it should also impose immediate design constraints on future studies [19,63].

### 6.3. Immediate Implications for Study Design

The framework also has immediate consequences for study design [19,63]. Biological standing should not be inferred from single-modality findings alone, whether genetic, imaging-based, molecular or computational [19,24,63,64]. New constructs should be benchmarked explicitly against generic severity, burden, chronicity and referral effects before stronger ontological claims are made [19,63]. Portability across ancestry, health-care setting and ascertainment frame should be treated as a component of validity rather than as a post hoc extension of initially local findings [74,76]. Proposed biotypes should be required to demonstrate clear advantages over simpler dimensional models in replication, prognostic increment and portability before being interpreted as disease entities [70,74,75]. Investigators proposing new transdiagnostic constructs should also report explicit boundary conditions, including what evidence would weaken or falsify the claim to disease-axis status [19,63].

In that application, “Met” indicates that the relevant criterion is supported by replicated and materially constraining evidence at the level discussed in the text; “Provisional” indicates meaningful but incomplete or portability-limited support; “Mixed” indicates uneven evidence across studies, levels, or ascertainment frames; and “Failed” indicates that the criterion is not yet credibly satisfied or is substantially undermined by negative evidence. Table 3 is intended as an illustrative application of the framework rather than as a definitive ontological adjudication of current constructs. It applies the framework to major current constructs, scoring each against the four core gatekeepers and at least one supporting/disciplining domain, and explicit boundary conditions, and it also assigns a provisional classification accordingly.

## 7. What Disease Axes Are Not

Several neighboring constructs in psychiatry are repeatedly over-read [19,63]. They may be descriptively useful, mechanistically suggestive, or clinically relevant, but that does not make them disease axes [63]. The issue is not whether they contain a signal. The issue is whether they organize illness in a way that warrants disease-level standing [19,63]. The distinction matters because constructs may summarize covariation, improve prediction, constrain mechanism, or assist stratification without thereby reaching the level of an organizing disease axis.

**Figure 2:**
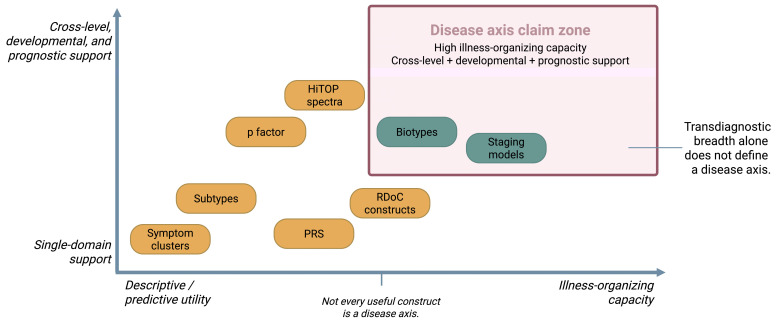
Disease axes and neighboring constructs: a conceptual map of organizing capacity versus evidential constraint. Disease-axis status requires more than utility, dimensionality, or mechanistic interest; it requires evidentially constrained illness-organizing force.

The conceptual boundary is important. A disease axis is not simply any construct that lies between syndrome and mechanism. Many such constructs are valuable, but they play different roles. Some mainly summarize covariation, some improve prediction, some constrain causal hypotheses, and some assist clinical stratification. The present argument reserves disease-axis status for constructs that do more than one of these things separately: they must begin to organize illness across levels, over time, and in relation to clinically consequential course. Figure 2 situates disease axes in relation to neighboring constructs, emphasizing that organizing capacity and evidential constraint are not interchangeable with descriptive breadth, predictive utility, or mechanistic interest.

Symptom clusters are descriptive groupings [61,63]. They may be clinically sensible and may occupy meaningful positions within a hierarchy, but they do not by themselves establish biology [61,63]. Descriptive subtypes are weaker still [19]. Most are carved retrospectively within diagnoses, often show limited longitudinal stability, and rarely demonstrate strong external validity [19,63]. Their biological relevance is commonly asserted after the clustering is complete rather than built into the construct from the start [19,63].

Endophenotypes deserve a more limited place in this discussion. The endophenotype program forced psychiatry to think seriously about validators rather than labels, and that was an important advance. But many proposed endophenotypes proved as pleiotropic and genetically complex as the syndromes they were meant to clarify. An intermediate phenotype may be biologically informative without organizing disease architecture.

The same caution applies to biomarkers without phenotypic anchoring. A robust molecular or imaging correlate may illuminate part of the biology of illness. But without stable anchoring to a dimension of liability, expression or progression, it is not disease architecture; it is a fragment of biology [19]. The inverse error is equally common: a statistically derived subgroup is labeled a biotype even when the biological measurements are only one ingredient in an unstable algorithm [70,74,75].

Machine-learning-derived biotypes illustrate the point sharply. Drysdale and colleagues reported depression biotypes defined by resting-state connectivity, linked to symptom patterns and differential treatment response. The study was influential because it appeared to bridge circuits, symptoms and intervention. But the subsequent replication effort by Dinga and colleagues failed to recover the critical statistical result, and broader methodological reviews have shown how sensitive this literature is to sample size, preprocessing, feature choices and external validation [74,75]. The problem is not simply a lack of replication. It is instability under perturbation of analytic choices [75]. Until such subtypes prove robust across methods, cohorts and outcomes, they should remain exploratory [70,74,75].

Generic severity continua are a subtler source of confusion. Large clinical datasets almost always contain a major axis of burden because more impaired patients tend to have more symptoms, more comorbidity and more dysfunction [61]. Such dimensions are clinically relevant and often prognostically useful. But cumulative burden is not the same thing as etiologic specificity. A general severity factor can be indispensable without being the best candidate for a biologically discriminable disease axis [71].

Cross-sectional data reduction creates the last common confusion [63,71]. A factor extracted from one time point may be mathematically coherent and still reveal little about the architecture of illness [63,71]. Psychiatric disease axes should organize onset, recurrence, divergence, remission and stage transition, not merely the geometry of one dataset [67]. Temporal anchoring is not optional [63,71].

## 8. What the Current Evidence Supports

Once descriptive success, strategic usefulness, and disease-level standing are separated, current constructs no longer appear as competing candidates of the same kind [19,63]. They differ not only in strength, but in evidential profile and epistemic role [19,63]. Some are best treated as summaries of psychopathology, some as useful instruments for prediction or stratification, and only some as plausible candidates for disease-axis status [19,63]. Table 3 presents a condensed comparative readout of the framework’s application to major current constructs, whereas Supplementary Table S1 provides the fuller decision matrix across the four core domains, the additional constraining domain, the presence or absence of stated boundary conditions, and the resulting provisional classification.

Under the present framework, middle-level spectra remain the strongest current candidates because they solve a problem that narrower syndromic categories and broader general factors solve less well [61]. They reduce some of the heterogeneity of diagnosis-bound constructs without collapsing psychopathology into an undifferentiated burden [61]. Their force lies not in dimensionality alone, but in the combination of replication, developmental coherence, and clinically relevant prognostic signal [19,61,63]. They are broad enough to overcome some of the heterogeneity of narrow syndromic diagnoses, yet not so broad that they dissolve psychopathology into one undifferentiated continuum [61]. At present, they offer the best compromise between biological plausibility and clinical recognizability and, therefore, emerge as the strongest candidates for provisional disease-axis status [61].

The p factor warrants a more divided judgment. It has clear descriptive importance: it captures broad liability, persistence, impairment, and burden, and it helps explain why diagnosis-bound biomarker findings have so often been nonspecific [19,61]. But under the present framework, its claim to disease-axis status remains limited by unresolved discriminability from nonspecific severity and by weak mechanistic constraint. For now, it is better treated as a summary of broad psychopathology than as a biological axis in its own right [61].

RDoC merits a different appraisal [64,78,79]. It has been among psychiatry’s most productive research frameworks because it reopened the study of core functional systems across diagnoses [79]. But RDoC constructs are better understood as mechanistic primitives than as disease axes [64]. Threat, reward, arousal, or working memory may intersect multiple disease axes rather than define one [79]. Their value lies in experimental tractability and mechanistic clarification, not in settling the unit of disease [64,79].

Biotypes and data-driven subtypes remain the most cautionary case [70,74,75]. Their promise is obvious, but psychiatry has repeatedly over-read clustering results [74,75]. Without robust replication across cohorts and analytic pipelines, developmental coherence, prognostic distinctiveness, and clear superiority to simpler dimensional models, they should remain exploratory rather than promoted disease entities [74,75]. Most current biotypes have not yet crossed that threshold [70,74,75].

Polygenic scores occupy another intermediate position. They are clearly biological and increasingly informative, but they do not currently organize clinical nosology. They support models of distributed and shared liability across disorders and may prove useful for risk architecture, prevention, and trial enrichment before they become useful for routine bedside decision-making. Yet their individual-level predictive power remains modest for most psychiatric purposes, and their portability across ancestries remains uneven. For now, they strengthen liability models, but they do not replace diagnosis and do not yet constitute disease axes in a clinically actionable sense.

Taken together, these judgments support a differentiated conclusion rather than a single victor. Middle-level spectra are presently the strongest candidates for provisional disease-axis status; the p factor remains descriptively central but ontologically unresolved; RDoC constructs clarify mechanism without defining disease units; biotypes remain exploratory; and polygenic scores are most useful at the level of liability architecture [19,61,63,64,70,74,75]. The value of the present framework is precisely that it allows these distinctions to be stated explicitly rather than blurred together under the general heading of dimensional psychiatry. The issue is therefore not which construct “wins”, but what kind of claim each construct can presently sustain.

## 9. The Strongest Objections

The history of recent psychiatric classification supports this caution. RDoC was one of the most ambitious attempts to redirect psychiatric research away from symptom-defined syndromes and toward dimensional constructs grounded in behavior, neural systems, and other biological levels of analysis. It was scientifically generative, but it did not become a broadly adopted clinical or nosological replacement for DSM or ICD. That limited uptake should not be dismissed as conservatism alone. It reflects a real translational gap between experimentally tractable constructs and clinically authoritative disease entities. RDoC clarified important systems of function, but clarification of function is not the same as the construction of a disease taxonomy.

DSM-5 illustrates the same problem from the opposite direction. Its development occurred during a period of substantial optimism that genetics, neuroimaging, cognitive neuroscience, and other biological measures might reshape psychiatric classification. In practice, however, available biomarkers were not sufficiently robust, specific, portable, or clinically actionable to serve as diagnostic criteria for most psychiatric disorders. This failure does not show that biological psychiatry failed; it shows that the evidential threshold for conferring disease status is high, especially when etiology remains unknown, probabilistic, or distributed across developmental, environmental, psychological, and biological levels.

This point applies not only to DSM and RDoC, but also to ICD, HiTOP, and p-factor approaches. ICD is more openly pragmatic and clinical-descriptive in orientation; DSM remains indispensable for communication, treatment planning, trials, and administration; HiTOP provides a strong empirical map of symptom covariation; and the p factor captures broad liability and burden. Yet none of these frameworks currently supplies a settled etiological taxonomy of psychiatric disease. Their claims differ in kind, but all remain constrained by the same fact: psychiatric phenomena are being classified in the absence of pathognomonic lesions, routine diagnostic biomarkers, and fully specified causal mechanisms.

The appropriate conclusion is therefore not that classification is futile, but that its claims must be graded. A construct may be clinically useful, structurally reproducible, prognostically informative, or biologically suggestive without thereby becoming a disease entity. This is precisely why the present framework emphasizes disciplined ontological promotion. It does not assume that psychiatry has already discovered biologically valid disease axes. It asks what evidence would be required before the field is justified in treating any dimensional or transdiagnostic construct as more than a useful descriptive or strategic tool.

The hardest objection is not methodological but ontological [19,63]. Psychiatry may never yield biologically privileged axes in any strong biological sense [19]. If liability is sufficiently distributed, developmentally plastic and environmentally contingent, then even well-validated dimensions may remain useful organizing abstractions rather than disease axes in any deeper sense [19,63].

A stronger challenge must also be faced [19,63]. Psychiatry may never yield disease axes that have the same status as more classically medical disease entities [19]. Liability may remain too distributed, developmentally contingent and environmentally modulated for any construct to achieve that status in a robust sense [19,63]. On that view, the main value of the framework would not be to secure a new psychiatric ontology, but to discipline what the field allows itself to infer from descriptive and biological findings [63]. That more modest aim is deliberate. In a field repeatedly damaged by reification, a framework may still be useful even if its main contribution is not to reveal final disease structure, but to make over-interpretation harder. Even if psychiatry ultimately settles for layered, pragmatically useful predictive frameworks rather than strongly privileged disease axes, the need for explicit promotion rules would remain [19,63].

That possibility has to be taken seriously. If anything, it raises the bar. The more uncertain the field is about whether any construct deserves disease-level standing in a strong sense, the greater the need for explicit rules about what evidence is and is not sufficient for ontological promotion [19]. The present framework does not assume that psychiatry has already identified such axes. It is meant to make the threshold for claiming them much harder to satisfy [19,63]. The limited nosological adoption of RDoC and the inability of DSM-5 to incorporate robust biological diagnostic markers should be read not as isolated failures, but as warnings against premature disease-level claims in a field where etiology is often unknown, distributed, and developmentally contingent.

A related objection is biological rather than strictly ontological: psychiatric liability may simply be too pleiotropic and too distributed for sharply bounded axes to emerge [66,73,80]. There is real force in that concern. Shared common variant liability, pleiotropic rare variants and overlapping molecular signatures all argue against simple one-disorder-one-mechanism models [45,66,73,80,81,82]. But distributed biology does not abolish structure. It changes the level at which structure should be sought [45,66,73]. The evidence currently points less toward sealed categories or one universal factor than toward several partially overlapping liabilities [45,66,81].

There is also a more familiar methodological danger [19,63]. Many dimensions may reflect severity, ascertainment or method variance more than substantive illness organization [19,63,74,76]. That is often true. Broad dimensions can be methodologically real and biologically shallow [19,63]. This is precisely why discriminant specificity and falsifiability are not optional extras in the present framework. A mature field should assume this danger from the outset rather than treating it as a hostile afterthought [19,63].

Neuroimaging raises a related concern [70,75,83,84]. Psychiatric imaging has produced many associations, but individual-level robustness has often been limited and translational utility modest [70,74,75,84]. That criticism is justified. The response is not to abandon circuit-level evidence, but to demote claims resting on imaging alone [75,84]. Imaging should count as one layer of convergence, never as a sovereign validator [70,75,84].

Much the same caution applies to psychiatric genetics [85,86,87]. Polygenic findings remain probabilistic and clinically underpowered for most routine uses [86,87]. Again, correct. But immediate bedside utility and explanatory relevance are not the same thing [85]. Even modestly predictive genetic findings can still reorganize nosological thinking by showing that current syndromic partitions align poorly with underlying liability [45,66,73,81,85]. Clinical modesty does not cancel conceptual force [85].

A more practical objection is that some statistically stable dimensions are clinically inert [19,63,71]. This is one of the clearest arguments against premature reification [19,63]. A factor that improves model fit but does not improve prognosis, stratification, staging or formulation may remain useful for description [19,63,71]. It has not yet earned biological standing [19,63].

The opposite risk must also be faced [45,61,66]. Broad transdiagnostic models may obscure distinctions that remain clinically essential [45,61]. This too is true. Patients are treated in particulars [61]. Shared liability does not make psychosis and mania interchangeable, nor compulsivity and anxiety, nor autism and schizophrenia [45,66,88]. Any viable axis-based psychiatry must therefore be layered rather than flattening [45,61]. Broad axes, syndromic presentations, stage and contextual modifiers should coexist [61,66].

Finally, there is a distributive objection that psychiatry can no longer afford to treat as secondary [89,90,91,92]. Sample bias and ancestry bias can make apparently biological constructs look more universal than they are [83,89,90,91,92]. A dimension that is unstable across populations, settings or health-care systems cannot simply be assumed to be a general disease axis [83,89,91,92]. Population portability is part of validity itself [83,92]. Any future axis-based nosology that ignores this point will reproduce the narrowness of the datasets from which it was built [89,91,92].

None of these objections invalidates the search for disease axes [19,63]. They do, however, rule out triumphalism [19]. Psychiatry should neither defend diagnosis as if biology had failed nor embrace dimensions as if statistics had already delivered disease architecture.

## 10. Clinical and Translational Implications

If biologically valid disease axes become the organizing target of psychiatric research, diagnosis will not disappear. But its status will change. DSM and ICD categories will remain necessary for communication, administration, service entry and many treatment decisions [19]. What should change is the assumption that they are the privileged biological unit [19,63].

In practical terms, this would not mean abandoning diagnosis at the bedside. It would mean that two patients who both meet criteria for major depression, for example, would no longer be treated as nosologically equivalent simply because they share a syndromic label. One might be understood primarily through a recurrent internalizing liability with preserved reality testing and high relapse risk; another through a broader liability pattern with emerging thought-disorder features, developmental instability, and different prognostic implications. The syndromic diagnosis would remain the entry point, but it would no longer be the sole organizing description of illness.

A more credible architecture would be layered [19,63,74]. It would begin with the syndromic presentation, because patients still present in recognizable clinical forms [19,63]. It would then add a profile on one or more empirically grounded disease axes, together with stage and trajectory, because where a person stands in the course of illness often matters more than static symptom count [61,70,75,76]. Context still matters: substance use, medical burden, adversity and functional decline do not become secondary because dimensions have entered the frame [74]. The gain would be a more realistic ordering of the problem. Diagnosis would retain its pragmatics, while biology and prognosis would move to a level they can more plausibly occupy [19,63,74]. Table 5 summarizes how this shift from diagnosis-bound to axis-informed reasoning changes the level at which clinically and scientifically meaningful inferences are made.

The nearest clinical gain is likely to be prognostic, not therapeutic [71,74]. Broad axes often forecast persistence, recurrence and impairment better than narrow diagnoses [71,74]. That matters. Disease axes may sharpen that forecast by integrating liability, stage and past course [61,74,76].

Treatment selection is a harder test [24,64]. Psychiatry should be plain about that. Axis-based models are not yet ready for routine bedside matching in most settings [64]. Their more credible near-term use lies in stratification and trial design: reducing phenotypic heterogeneity, enriching samples, and identifying subgroups in whom particular mechanisms are more likely to matter [24,64,73]. For now, precision psychiatry is more convincing as better stratification than as bespoke treatment assignment [24,64].

Prevention and early intervention may prove more tractable [61,70,75,76]. Youth services have already moved toward transdiagnostic staging because early psychopathology is often pluripotent and syndromic diagnosis performs poorly at that stage [70,75]. Disease axes could strengthen that work by identifying which early constellations of symptoms and liabilities are associated with persistence, diversification or progression [61,75,76]. In that respect, axes may turn out to be more useful for risk architecture than for final diagnosis [61,70,76].

Clinical reasoning may improve as well, provided psychiatry does not mistake scoring for understanding [19,63]. Experienced clinicians already think dimensionally, whether or not they use that language explicitly [19,63,66]. A better axis framework would not replace that reasoning. It would discipline it [19,63]. But an axis is not a formulation, and a score is not a patient [63]. The point is not to replace psychopathology with dashboards. It is to align research constructs more closely with the way illness actually unfolds [19,63].

There are risks, and they are not trivial. Axis language can invite abstraction, overconfidence and neglect of phenomenological detail [19,63]. It can also generate a fresh reification: the belief that a dimensional score is automatically closer to truth than a clinical syndrome [19,63]. Psychiatry has made that mistake before, only with different units [19]. The corrective is unchanged. Concepts must remain accountable to outcome, mechanism, development and clinical use. The point is not that such distinctions are already ready for routine codification, but that they illustrate how layered formulations may become more clinically informative than syndromic equivalence alone.

## 11. Limitations

Several limitations should be acknowledged. First, this article is a conceptual synthesis rather than a systematic review, scoping review, or meta-analysis. The literature search was targeted and purposive, and was used to support framework development rather than to provide exhaustive evidence retrieval, formal quality grading, or quantitative synthesis. The comparative judgments offered in the tables should therefore be read as structured interpretive appraisals, not as results of a formal evidence-ranking procedure.

Second, the proposed framework is not intended to function as a mechanical diagnostic algorithm or as a definitive ontology of psychiatric disease. Its purpose is more limited: to discipline the inferential step from descriptive structure or predictive utility toward stronger disease-level claims. The framework may help investigators decide when disease-axis language is defensible, but it cannot determine by itself what psychiatric disease architecture ultimately is.

Third, the ratings assigned to current constructs remain provisional and dependent on the present state of evidence. Constructs such as the HiTOP internalizing spectrum, the p factor, RDoC domains, polygenic scores, clinical staging models, and data-driven biotypes are rapidly evolving. Their evidential status may change as larger, more diverse, longitudinal, multimodal, and clinically anchored studies become available.

Fourth, the framework necessarily simplifies a complex validation landscape. Replication, developmental coherence, prognostic increment, discriminability from nonspecific severity, mechanistic constraint, clinical leverage, and falsifiability are analytically separable, but in practice, they often interact. A construct may show strength in one domain and weakness in another, and expert judgment will still be required when evidence is uneven.

Finally, clinical implementation remains premature for most proposed disease axes. The framework is best understood as a research and reporting tool at this stage. It may support better construct development, study design, trial enrichment, and prognostic stratification, but it should not be taken to imply that dimensional disease-axis models are ready to replace established clinical diagnoses in routine care.

## 12. Conclusions

Psychiatry has been right to move beyond the fiction that DSM and ICD categories are final biological entities. But the field has made a reciprocal mistake: it has too often treated replicated latent structure as if replication itself conferred disease-level standing. The central question is therefore not whether psychopathology is dimensional, but what justifies stronger claims that a construct should be interpreted as part of candidate disease architecture rather than as a descriptive or predictive model. The argument advanced here is that psychiatry now needs an explicit rule of ontological promotion, and that such a rule should be cumulative, falsifiable and clinically consequential. A biologically credible psychiatric axis should therefore earn promotion only through cumulative evidence across levels, development, prognosis, mechanism, specificity, clinical leverage and falsifiability. By that standard, some middle-level transdiagnostic spectra and some cross-disorder liabilities deserve serious consideration as candidate disease axes, whereas other constructs remain descriptive, strategically useful, or unsupported as candidate disease axes. This does not mean that all well-validated transdiagnostic constructs qualify as disease axes, but only that some may begin to assume that role when they organize illness rather than merely summarize risk, mechanism, or prediction. The future is neither a naive defense of diagnosis nor a simple replacement of diagnosis by one master factor. It is a layered nosology in which syndromic presentation, disease axes, stage and context are modeled together. Psychiatry will progress not by choosing dimensions over categories, but by making any stronger disease-axis claim answerable to disciplined rules of ontological promotion.

As a practical implication, future investigators proposing a new dimensional or transdiagnostic construct should treat this framework as a structured reporting checklist rather than as a rhetorical aspiration. At minimum, they should specify what the construct is intended to explain; distinguish whether the claim is descriptive, prognostic, mechanistic, or disease-level; report evidence for the four core gatekeepers; identify which supporting/disciplining domains are present; benchmark the construct against diagnosis, nonspecific severity, chronicity, and total symptom burden; and state explicit boundary conditions, including findings that would weaken or falsify the claim. A proposed construct should not be presented as a candidate disease axis merely because it is statistically stable, biologically associated, or clinically interesting. The stronger claim should be reserved for constructs that remain portable, developmentally intelligible, prognostically informative, sufficiently discriminable from nonspecific burden, and exposed to disconfirmation.

## Figures and Tables

**Table 1:** Alignment of the eight evidential domains with the Section 5 framework.

Section	Evidential Domain	Role Within the Framework	Operational Question
Section 5.1	Cross-level convergence	Supporting/ disciplining domain	Does the construct show patterned convergence across clinical, behavioral, biological, and longitudinal levels rather than isolated correlates?
Section 5.2	Replication across cohorts, ascertainment, and methods	Core gatekeeper	Does the construct remain recognizable across independent cohorts, ascertainment frames, instruments, and analytic pipelines?
Section 5.3	Developmental coherence	Core gatekeeper	Does the construct show an intelligible developmental profile, including antecedents, age-sensitive expression, persistence, transformation, or progression?
Section 5.4	Prognostic increment beyond diagnosis and nonspecific severity	Core gatekeeper	Does the construct improve prediction of clinically consequential outcomes beyond diagnosis, symptom burden, and generic severity?
Section 5.5	Mechanistic constraint	Supporting/ disciplining domain	Does the construct narrow plausible causal or mechanistic models rather than merely accumulating biological associations?
Section 5.6	Discriminability from nonspecific severity	Core gatekeeper	Does the construct remain interpretable after adjustment for generic distress, chronicity, impairment, referral intensity, or total symptom burden?
Section 5.7	Clinical leverage	Supporting/disciplining domain	Does the construct change formulation, stratification, prognosis, monitoring, prevention, trial enrichment, or treatment-relevant inference?
Section 5.8	Falsifiability and boundary conditions	Supporting/disciplining domain	Are the boundary conditions specified, including findings that would weaken, restrict, or falsify the disease-axis claim?

Note: The terminology in this table matches Section 5 subheadings. Core gatekeepers define the minimum evidential requirements for considering disease-axis promotion. Supporting/disciplining domains strengthen, constrain, or expose the claim to disconfirmation, but do not substitute for the core gatekeepers.

**Table 2:** Operational thresholds for the four core gatekeepers in candidate disease-axis evaluation.

Core Gatekeeper	Met	Provisional	Failed
Replication across cohorts, ascertainment, and methods	Construct is reproduced across independent cohorts, different ascertainment frames, measurement strategies, and analytic pipelines without major loss of interpretive meaning.	Construct is reproduced only in closely related datasets, similar instruments, or narrow ascertainment settings.	Construct is unstable across methods, settings, populations, or analytic pipelines, or changes substantially under modest methodological perturbation.
Developmental coherence	Construct shows a recognizable developmental profile, including antecedents, age-sensitive expression, and lawful persistence, transformation, or progression.	Construct shows limited longitudinal support, short-term stability, or cross-sectional age gradients without a clearly established developmental trajectory.	Construct lacks a coherent developmental profile or does not survive longitudinal examination.
Prognostic increment beyond diagnosis and nonspecific severity	Construct meaningfully improves prediction of clinically consequential outcomes beyond diagnosis, symptom burden, and generic severity.	Construct shows small, inconsistent, or statistically detectable but clinically limited prognostic gain.	Construct adds no meaningful prognostic information beyond existing diagnostic and severity-based models.
Discriminability from nonspecific severity	Construct remains interpretable after adjustment for generic distress, chronicity, impairment, referral intensity, or total symptom burden.	Construct shows some evidence of specificity, but residual confounding by nonspecific severity, burden, or chronicity remains plausible.	Construct largely collapses into generic distress, cumulative burden, chronicity, or overall impairment once these factors are modeled explicitly.

Note: Met, provisional, and failed refer to the current evidential status of a promotion claim, not to definitive ontological truth. These gatekeepers are intended to prevent premature disease-axis claims when a construct is insufficiently replicated, developmentally incoherent, prognostically inert, or indistinguishable from nonspecific severity.

**Table 3:** Illustrative comparative appraisal of major current constructs under the proposed evidential thresholds.

Construct	Replication	Developmental Coherence	Prognostic Increment	Discriminability from Nonspecific Severity	Supporting/ Disciplining Evidence	Dominant Limiting Factor/Dominant Strength	Provisional Classification
Middle-level spectra (e.g., internalizing, externalizing, thought disorder)	Met	Met	Met	Provisional	Partial cross-level convergence, including genomic overlap and recognizable phenotypic structure	Strongest current balance between breadth, discriminability, and prognostic coherence without collapsing into undifferentiated burden	Strongest current candidates for provisional disease-axis status
p factor/general psychopathology factor	Met	Provisional	Met	Mixed	Broad liability signal, persistence, and burden capture across disorders	Broad liability signal remains difficult to disentangle from nonspecific severity, chronicity, and cumulative burden	Descriptively central, but ontologically unresolved
RDoC constructs	Mixed	Mixed	Mixed	Mixed	Mechanistic and experimental tractability across functional domains	Mechanistic usefulness exceeds disease-organizing specificity; these constructs clarify processes more readily than disease architecture	Mechanistically informative constructs rather than disease axes
Biotypes/data-driven subtypes	Provisional	Failed	Mixed	Mixed	Biological and circuit-level promise in selected studies	Mechanistic promise is undermined by weak portability, unstable replication, and limited developmental or prognostic durability	Exploratory partitions, not promoted disease entities
Polygenic scores (PRS)	Met	Provisional	Provisional	Mixed	Genomic constraint and cross-disorder liability signal	Liability signal is real, but clinical interpretability and portability remain too limited for disease-axis standing	Strategically useful for liability architecture, but not disease axes in a clinically actionable sense
Clinical staging/dynamic transdiagnostic progression models	Provisional	Met	Met	Provisional	Dynamic relevance to progression, persistence, and transition	Strong temporal and prognostic relevance, but replication and portability remain less secure than required for stronger promotion claims	Promising strategically useful constructs; possible future candidates if replication and portability strengthen

Note: Ratings are intentionally interpretive rather than algorithmic and are offered to show how the framework may structure comparison across constructs. They should be read as provisional comparative judgments based on the criteria defined in the main text, not as definitive ontological classifications.

**Table 4:** Worked application of the four core gatekeepers to two existing constructs.

Construct	Replication	Developmental Coherence	Prognostic Increment	Discriminability from Nonspecific Severity	Provisional Interpretation
HiTOP internalizing spectrum	Met	Met	Met/Provisional	Provisional	Stronger provisional candidate for disease-axis status than highly global constructs, provided that it retains specificity after severity and chronicity are modeled explicitly.
General psychopathology factor/p factor	Met	Provisional	Met	Mixed/Provisional	Descriptively central and prognostically informative, but ontologically unresolved because much of its signal may reflect nonspecific burden, chronicity, impairment, or cumulative adversity.

Note: Ratings are illustrative and provisional. They are intended to show how the four core gatekeepers can be applied in practice, not to provide definitive ontological classifications.

**Table 5:** How an axis-based framework changes clinical and research inference beyond diagnosis alone.

Level	Traditional Diagnosis-Bound Inference	Axis-Informed Inference	Practical Consequence
Case formulation	Patients meeting the same syndromic criteria are treated as nosologically equivalent, with differences handled mainly as severity, comorbidity, or context	The same syndromic presentation may reflect different liabilities, patterns of expression, and trajectories of progression	Layered formulation beyond checklist equivalence; greater precision in describing what kind of illness process is being treated
Prognosis	Prognosis is inferred chiefly from diagnosis, baseline severity, and prior episodes	Prognosis is informed by spectrum position, developmental coherence, stage, and progression signal in addition to diagnosis	Better stratification of monitoring intensity, follow-up planning, and relapse prevention
Trial enrichment	Recruitment is organized around broad syndromic categories that often contain marked internal heterogeneity	Recruitment is organized around more coherent liabilities or progression-relevant dimensions within and across syndromic categories	Reduced phenotypic noise, stronger signal detection, and more interpretable treatment-response heterogeneity
Early intervention	Early symptoms are interpreted through unstable or provisional syndromic labels that may change over time	Early presentations are understood in terms of pluripotent liabilities, developmental trajectories, and risk of persistence or diversification	More credible preventive targeting and less overcommitment to premature categorical labeling
Staging	Stage is often secondary to diagnosis and commonly inferred indirectly from chronicity or service use	Stage is treated as an organizing feature of illness in its own right, linked to progression, transition, and persistence	More clinically informative distinction between current presentation and underlying trajectory of illness
Mechanistic research	Mechanistic studies are anchored primarily to heterogeneous diagnostic syndromes	Mechanistic studies are anchored to constructs that better organize liability, expression, or progression across traditional categories	Better alignment between biological investigation and clinically meaningful illness structure
Biomarker research	Biomarkers are tested against broad syndromic targets with high internal heterogeneity and limited biological tractability	Biomarkers are tested against narrower organizing liabilities or progression-relevant dimensions rather than diagnosis alone	Stronger target specification, improved interpretability, and less risk of syndrome-bound false negatives
Clinical decision-making	Diagnostic assignment remains the dominant organizer of treatment planning, with dimensions used mainly descriptively	Diagnosis remains the entry point, but dimensional and staging information modifies how risk, course, and likely burden are interpreted	More differentiated care planning without abandoning the pragmatic value of diagnosis
Nosological reasoning	A construct is often taken seriously once it is reliable, predictive, or biologically suggestive in isolation	A construct is granted stronger standing only when it survives replication, developmental, prognostic, and specificity thresholds	Greater discipline in moving from descriptive success to stronger disease-level claims

Note: The table is intended to show how the proposed framework changes the level at which clinically and scientifically meaningful inferences are made. It does not imply that diagnosis becomes obsolete; rather, it shows how diagnosis may cease to function as the sole organizing description of illness.

## Data Availability

No new data were created or analyzed in this study.

## Supplementary Materials

The following supporting information can be downloaded at: https://www.mdpi.com/article/10.3390/jcm15124830/s1, Supplementary Table S1: Full decision matrix for illustrative comparative appraisal of major current constructs under the proposed evidential thresholds.

## Abbreviations

The following abbreviations are used in this manuscript:

DSM	Diagnostic and Statistical Manual of Mental Disorders
DSM-III	Diagnostic and Statistical Manual of Mental Disorders, Third Edition
ICD	International Classification of Diseases
HiTOP	Hierarchical Taxonomy of Psychopathology
MEDLINE	Medical Literature Analysis and Retrieval System Online
PRISMA	Preferred Reporting Items for Systematic Reviews and Meta-Analyses
PRS	Polygenic risk scores
RDoC	Research Domain Criteria
p factor	General psychopathology factor
