# Supplementary material for: Beyond DSM Categories: Criteria for Biologically Valid Disease Axes in Psychiatry
Source: J Clin Med. 2026 Jun 22;15(12):4830. doi: 10.3390/jcm15124830 (PMC13301616; doi:10.3390/jcm15124830)
Supplement: Supplementary file 1 [file jcm-15-04830-s001.zip › jcm-4331559-supplementary.pdf]

Supplementary Table S1. Full decision matrix for illustrative comparative appraisal of major current constructs under the proposed evidential thresholds

Construct	Replication	Developmental coherence	Prognostic increment	Discriminability from nonspecific severity	Additional constraining domain(s)	Current disqualifying condition(s)	Brief rationale for comparative judgment	Provisional classification
**Middle-level spectra (e.g., internalizing, externalizing, thought disorder)**	Met	Met	Met	Provisional	Partial cross-level convergence, including genomic overlap and recognizable phenotypic structure	Residual concern that some variance still reflects broad burden rather than fully discriminable disease organization	These spectra currently offer the best balance between reproducibility, developmental intelligibility, and clinically meaningful prognostic signal. They are broader and more stable than narrow syndromic categories, but more discriminating than fully general liability constructs.	Strongest current candidates for provisional disease-axis status
**p factor / general psychopathology factor**	Met	Provisional	Met	Mixed	Broad liability signal, persistence, and burden capture across disorders	Unresolved specificity relative to nonspecific severity, chronicity, and cumulative burden; limited mechanistic constraint	The p factor remains descriptively indispensable and clinically informative at the level of broad burden, but its claim to disease-axis standing is weakened by uncertainty over whether it reflects specific shared pathophysiology or predominantly global severity.	Descriptively central, but ontologically unresolved
**RDoC constructs**	Mixed	Mixed	Mixed	Mixed	Mechanistic and experimental tractability across functional domains	Lack of stable disease-organizing specificity; variable relevance to prognosis, development, and discriminability at the disease-axis level	RDoC constructs are highly useful for experimental and mechanistic clarification, but they function more naturally as functional or process-based primitives than as organizing axes of illness. Their main strength lies in tractability rather than disease-level integration.	Mechanistically informative constructs rather than disease axes
**Biotypes / data-driven subtypes**	Provisional	Failed	Mixed	Mixed	Biological and circuit-level promise in selected studies	Weak portability across cohorts and pipelines; instability under methodological perturbation; limited	These constructs remain promising as exploratory partitions and for mechanism-oriented work, but current evidence does not yet justify stronger disease-axis claims because stability across	Exploratory partitions, not promoted disease entities

						developmental durability	settings, time, and outcomes remains insufficient.	
**Polygenic scores (PRS)**	Met	Provisional	Provisional	Mixed	Genomic constraint and cross-disorder liability signal	Limited portability across ancestries; modest clinical interpretability and predictive utility at the individual level	PRS clearly support distributed liability architecture and strengthen models of cross-disorder risk, but they do not yet organize clinical illness strongly enough to function as disease axes in a clinically actionable sense.	Strategically useful for liability architecture, but not disease axes in a clinically actionable sense
**Clinical staging / dynamic transdiagnostic progression models**	Provisional	Met	Met	Provisional	Dynamic relevance to persistence, transition, and progression	Cross-cohort portability and replication remain less secure than needed for stronger promotion claims	These models capture something static syndromic diagnosis often misses: clinically meaningful structure may lie in progression and transition rather than symptom profile alone. Their main promise lies in temporal and prognostic organization, but replication across settings remains the chief bottleneck.	Promising strategically useful constructs; possible future candidates if replication and portability strengthen

Note. *Ratings are intentionally interpretive rather than algorithmic and are provided to make the grounds of comparison explicit. They should be read as structured comparative judgments under the evidential thresholds defined in the main text, not as definitive ontological classifications.*
